# GIST: Correlation of risk classifications and outcome

**DOI:** 10.25122/jml-2021-0110

**Published:** 2022-08

**Authors:** Sabine Kersting, Monika Silvia Janot-Matuschek, Carina Schnitzler, Daniel Enrique Chourio Barboza, Waldemar Uhl, Ulrich Mittelkötter

**Affiliations:** 1 Department of General Surgery, Christliches Klinikum Unna Mitte, Unna, Germany; 2 Department of General Surgery, St. Josef-Hospital Bochum, Ruhr-University Bochum, Bochum, Germany

**Keywords:** Gastrointestinal stromal tumor (GIST), secondary neoplasia, recurrence risk, metastasis risk, proliferation rate, CD – cluster of differentiation, DOG – discovered on gastrointestinal stromal tumors protein, GIST – gastrointestinal stromal tumor, HPF – high-power field, IPMN – intraductal papillary mucinous neoplasm, KIT – stem cell factor receptor, tyrosine kinase, MIB – made in borstel, NIH – National Institutes of Health, PDGFRA – platelet-derived growth factor, TNM – tumor nodes metastases, UICC – Union Internationale Contre le Cancer

## Abstract

In clinical practice, there are often discrepancies between the oncological prognosis of gastrointestinal stromal tumors (GIST) and the actual clinical course. This study aimed to check with our collective how reliably the current classifications (Miettinen, Fletcher) predict the prognosis of GIST and to evaluate whether an extension of the classifications by the parameter proliferation activity could make sense. This prospective study enrolled 58 patients who underwent surgery on GIST from 01/2006 to 12/2016. The postoperative course (curation, recurrence, progress) was correlated with the identified risk classification and the proliferative activity. Coincidences with other tumors were strikingly common in patients with GIST (43%). Based on the risk group assignment of GIST, no assessment of the probability of the occurrence of second neoplasia could be derived. Individual patients were under- or over-graduated concerning the assessment of biological behavior based on the standard risk classifications. The inclusion of proliferative activity did not allow for a more precise predictive power - neither to the risk of recurrence and metastasis nor to the development of a second neoplasia. The study showed that there is currently no parameter or logarithm that reliably predicts the biological behavior of GIST. Due to the frequency of coincidence of second neoplasia and (rare) distant metastases, for everyday clinical practice, appropriate staging diagnostic and regular follow-up care should also be used for benign GIST.

## INTRODUCTION

Gastrointestinal stromal tumors (GIST) are mesenchymal tumors of the gastrointestinal tract. They grow submucosally and, in contrast to carcinomas or sarcomas, show malignant behavior with infiltrative growth, peritoneal seeding, or haematogenic metastasis in only 20–30% [1]. Various classifications have been published based on markers such as tumor size, location, and mitotic rate and aim to assess the tumor aggressiveness and prognosis of GIST [2–7].

GIST were first described as a separate tumor entity in 1998 [8]. The incidence is 0.3–2 cases per 100000 population. However, it can be assumed that there is a high number of unreported cases of small tumors that remain asymptomatic and are diagnosed as secondary findings [9]. GIST are often asymptomatic until they reach a displacing size. They are accidentally discovered during imaging examinations or an operative intervention [10]. With a share of 55%, men are slightly more frequently affected than women. The mean age is around 60 years [11]. The literature describes a high coincidence rate with secondary carcinomas, which is reported to be up to 43% [12].

The immunohistochemically detectable expression of receptor tyrosine kinase (KIT) [cluster of differentiation (CD) 117] and discovered on gastrointestinal stromal tumors protein (DOG) 1 [13] is pathognomonic for over 95% of all GIST. GIST occur in 60–70% in the stomach. Localization in the small intestine is found in 25–35%, in the colon and rectum in 5%, and in the esophagus in 2% of the cases. The location of the tumor is important in the assessment of dignity. Tumors in the small intestine are more frequently malignant than in the stomach [14]. Malignant GIST have a metastasis rate greater than 80% [11]. Resection is indicated for GIST that have progressively increased in size and are more than 2 cm in size [15]. The aim is the surgical removal of the tumor according to oncological criteria. A tumor rupture must be avoided as a matter of urgency since a curative approach is only given by an R0 resection [16, 17].

90% of GIST have activating mutations in the proto-oncogenes that code for the receptor tyrosine kinases KIT or platelet-derived growth factor (PDGFRA) [18 ,19]. The type of mutation has an impact on the prognosis as well as on the response to therapy and can therefore be used to determine further therapy concepts [18, 19]. Tyrosine kinase inhibitors (imatinib, sunitinib, regorafenib) are used in metastatic, perforated, and non-resectable GIST and if there is a significant risk of recurrence after resection of GIST [17, 20–22].

This study was based on the observation that in everyday clinical practice there are often discrepancies between the oncological assessment of GIST (based on the above-mentioned classifications) and the actual clinical course (recurrence rate, distant metastasis, secondary tumors). It is possible that the current classifications need to be supplemented with additional parameters in order to be more effective in practice, *i.e*., to assess the actual prognosis even more precisely and thus provide the patient with optimal therapy or follow-up care. This study aimed to investigate the outcome of patients with GIST and the association with secondary malignancies. The current classifications of the prognosis assessment were verified based on our own patient collective, and it was evaluated whether an extension of the classifications to include the proliferation activity as a further histopathological parameter makes sense.

## Material and Methods

### Data acquisition

This prospective study recorded all patients who were operated for a GIST in the Department of General Surgery, Christliches Klinikum Unna Mitte (n=37) and in the Department of General Surgery, St. Josef-Hospital, Ruhr-University Bochum (n=21) between January 2006 and December 2016. The histological examinations of the specimens were carried out by the Institute for Pathology at the Ruhr-University Bochum.

The following variables were examined: age, gender, type of primary intervention, complications, secondary malignancies, and mortality. In addition to tumor size and location, histopathological tumor characteristics [number of mitoses, antibody positivity to CD 117, made in borstel (MIB) 1 proliferation rate] were recorded. The staging and risk stratification was carried out according to the criteria of Fletcher [2] (Table 1 A) and Miettinen and Lasota [6] (Table 1 B). An analysis for the presence of mutations in the KIT or PDGFRA gene has been performed regularly since 2010 in patients whose GIST correlated with a rather unfavorable prognosis according to these classifications, as well as in patients with metastatic GIST.

**Table 1A:** Risk classification of gastrointestinal stromal tumors (GIST) according to Fletcher *et al*. 2002 [2].

Risk group	Tumor size (cm)	Mitoses/50 HPF*
**Very low**	<2	<5
**Low**	2–5	<5
**Intermediate**	<5	6–10
	5–10	<5
**High risk**	>5	>5
	>10	Any number
	Any size	>10

* – high power field.

**Table 1B:** Risk classification of gastrointestinal stromal tumors (GIST) according to Miettinen and Lasota 2006 [6].

Size (cm)	Mitoses/50 HPF*	Stomach	Duodenum	Jejunum/Ileum	Rectum
**≤2**	-	-	-	-	-
**>2/≤5**	≤5	-	Low	Low	Low
**>5/≤10**	-	Low	Intermediate	-	-
**>10**	-	Intermediate	High	High	High
**≤2**	-	-	High	-	High
**>2/≤5**	>5	Intermediate	High	High	High
**>5/≤10**	-	High	High	High	High
**>10**	-	High	High	High	High

* – high power field.

Also, the operated patients were contacted again for a follow-up evaluation of the further course of the disease (follow-up time 2–84 months; mean 42 months). The postoperative course (curation, relapse, progress) was correlated with the determined risk classification and the proliferation rate as a parameter of the proliferation activity. The postoperative course (curation, relapse, progress) was correlated with the determined risk classification and the proliferation rate, which reflects the proliferation activity of the GIST.

### Evaluation of the data/statistics

The correlation of the tumor proliferation rate with the occurrence of second malignancies was evaluated using a four-field test at a significance level of 0.05 (software: SPSS statistics R version 3.2.2.). A univariate and multivariate analysis was performed to evaluate parameters influencing patient outcome in terms of death from GIST and recurrence. Parameters relevant to the outcome are displayed using Kaplan Meier curves.

## Results

### Patients

The gender ratio in the patient collective was almost balanced: 27 women (47%) and 31 men (53%). The age was between 31 and 86 years (median 65 years). A peak was documented in the range between 66 and 85 years. 52 of the patients were older than 50 years (90%), six of the patients were younger than 50 years (10%).

**Figure 1:**
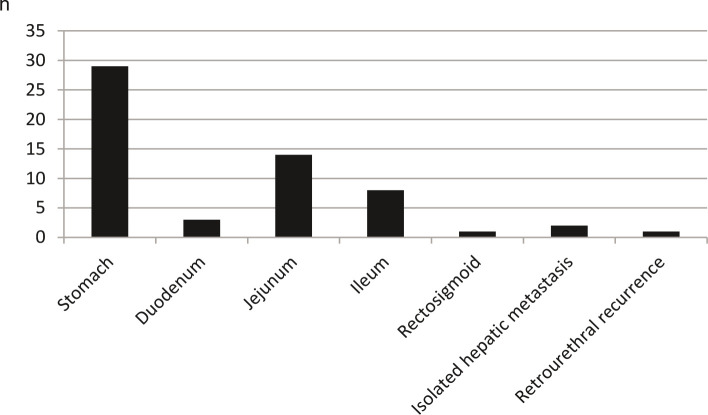
Localization of gastrointestinal stromal tumors (GIST).

The different locations of GIST are shown in Figure 1. 26 patients (45%) were operated because of the endoscopic suspicion of GIST. In 22 patients (38%), the diagnosis of GIST was made incidentally during other operations. Eight patients (14%) underwent surgery for unexplained upper gastrointestinal bleeding. In two patients (3%), the operation was performed because of distant metastasis (hepatic metastasis after resection of a colon GIST 10 years earlier and retrourethral metastasis after resection of a gastric GIST 8 years earlier).

### Surgical procedure

**Figure 2:**
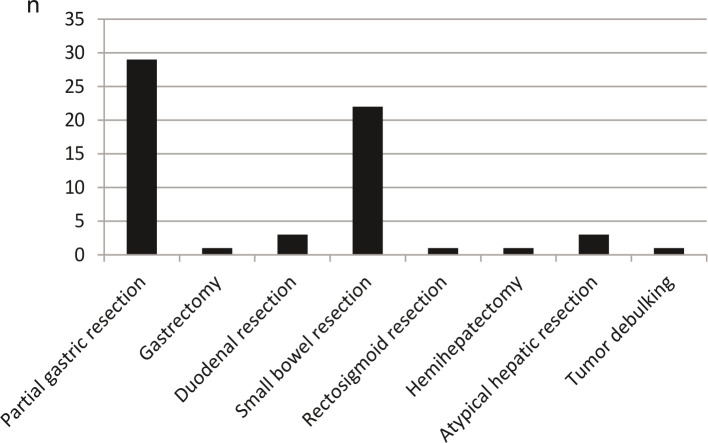
Surgical procedure for gastrointestinal stromal tumors (GIST). In one patient, a partial hepatectomy was performed in addition to resection of the primary in the stomach. In another patient, partial hepatectomy and partial gastric resection were performed in addition to resection of the jejunal GIST. Therefore, the total number of interventions was n=61.

R0 resection of GIST succeeded in 56 patients (97%) (Figure 2). In two patients, no R0 resection could be performed. This included a patient with GIST of the small intestine who presented with disseminated peritoneal carcinosis and bladder infiltration. It was suspected preoperatively that it was a gynecological tumor originating in the ovary. The second patient was the above-mentioned patient with retrourethral metastasis and infiltration of the bladder.

### Complications

Four patients (7%) experienced non-GIST disease-related complications. These were infection of a central venous catheter after laparoscopic gastric wedge resection, a hematoma not requiring revision in the paracolic gutter after sigmoid resection, a deep vein thrombosis with consecutive pulmonary embolism as well as pneumonia after gastrectomy, an infected lymph fistula, and a deep vein thrombosis after pancreatectomy.

Two patients presented complications associated with GIST disease. The above-mentioned patient with hepatic metastasis of a colon GIST died after hemihepatectomy. There was bleeding with hemorrhagic shock postoperatively. In addition, she developed severe necrotizing pancreatitis. Another patient developed an anastomotic leak and gastric entrance obstruction following gastric resection.

No GIST-specific surgical complications occurred among the patients whose GIST diagnosis was an incidental finding during another operation.

### Presence of secondary tumors

Of the 58 patients, 21 (36%) had already died at the time of the follow-up survey. There were six women and 15 men. Of these 21 patients, eight died of second malignancies.

25 (43%) of all examined patients fell ill synchronously or metachronically with a second benign or malignant tumor. Six patients (10%) had the following benign tumors synchronously: lipoma, Recklinghausen's disease with multiple neurofibromas, liver hemangiomas (2 patients), mucinous cystadenoma, and intraductal papillary mucinous neoplasm (IPMN) of the pancreas. Synchronous or metachronous malignancies were found in 20 patients (34%). There were three pancreatic carcinomas, two neuroendocrine pancreatic tumors, and one distal bile duct carcinoma. Carcinomas of the gastrointestinal tract were diagnosed in seven patients: one esophageal carcinoma, three gastric carcinomas, two colon carcinomas (one patient also suffered from B non-Hodgkin's lymphoma and a spinocellular scalp carcinoma), and one rectal carcinoma. Three patients had a history of malignant melanoma, and two patients were diagnosed with prostate cancer years before the GIST (one patient with prostate cancer also had the pancreas IPMN mentioned above). One patient developed metachronous breast cancer. Another patient had a history of undifferentiated lymphoma.

### Risk classification of our patient collective according to Fletcher and Miettinen

**Figure 3:**
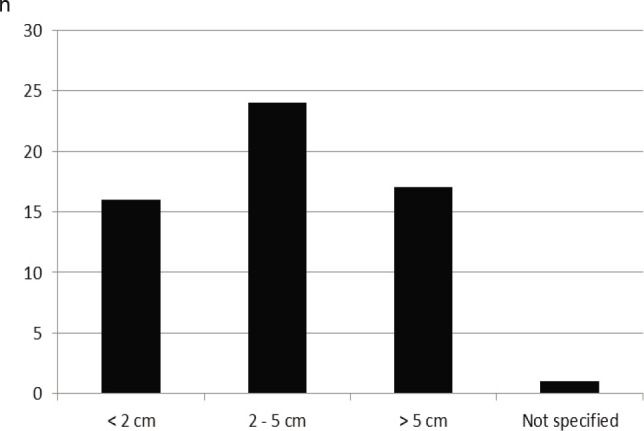
Size distribution of gastrointestinal stromal tumors (GIST).

**Figure 4:**
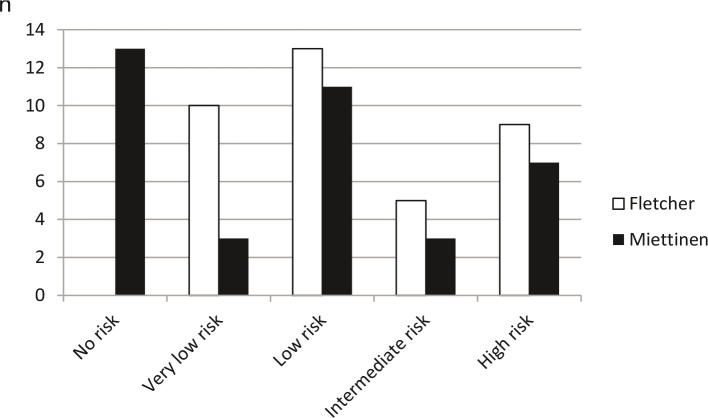
Allocation of our patient collective to the classifications according to Fletcher and Miettinen. A mitotic rate was given in the pathological findings of 37 patients so that an assignment to the classifications could be made for these patients.

The tumor size varied between 0.1 cm and 13 cm (mean 3.2 cm) (Figure 3). The tumor cells of all patients were positive for the staining of the KIT receptor tyrosine kinase CD 117. 37 of the patients could be assigned to the classifications according to Fletcher or Miettinen (Figure 4). A mitotic rate was given in the histological findings of these patients.

The risk classifications, according to Fletcher and Miettinen, would have led to a clear undergraduation in the case of a patient with liver metastasis already present at the initial diagnosis of gastric GIST. According to Fletcher and Miettinen, the primary tumor in the stomach with a size of 3 cm and a mitotic rate of 1/50 high-power field (HPF) would have been assigned to the low-risk group. However, this does not correspond to the actual biological behavior of the tumor, which had already metastasized into the liver at the time of the operation and the initial diagnosis (Table 2).

**Table 2:** Metastatic gastrointestinal stromal tumors (GIST): Histopathological findings, tumor size, risk classification, outcome.

Localization of metastasis	Liver	Liver	Liver	Peritoneum	Liver	Liver	Greater ommentum	Retrourethral
**Time of metastasis**	Intraoperative findings	Intraoperative findings	Intraoperative findings	Intraoperative findings	In the course of the follow-up	Intraoperative findings	Intraoperative findings	Intraoperative findings
**Localization of GIST**	Transverse colon	Not named	Stomach	Ileum	Jejunum	Jejunum	Stomach	Stomach
**Size of GIST**	Not named	Not named	3 cm	13 cm	10 cm	8.5 cm	25 cm	Not named
**Proliferation rate**	10%	70%	20%	Not named	Not named	Not named	Not named	15%
**Mitoses/50 HPF ^†^**	Not named	Not named	1/50 HPF	Not named	Not named	8/50 HPF	36/50 HPF	18/50 HPF
**Classification according to Fletcher**	*	*	Low risk	High risk	High risk	High risk	High risk	High risk
**Classification according to Mittienen**	*	*	Low risk	High risk	High risk	High risk	High risk	High risk
**Outcome**	Deceased on hemorrhagic schock	Deceased on pancreatic cancer	Deceased on tumor progression	Deceased on pancreatic carcinoma, but also tumor progression	Deceased on tumor progression	Deceased on tumor progression	Remission	Deceased on tumor progression

* – Assignment to the classifications according to Fletcher and Miettinen is not possible if the mitotic rate is missing. ^†^ – high power field.

### Results of the mutation analysis

Mutation analysis was carried out in nine patients (Table 3). These were patients operated on for a metastatic GIST or a GIST between 2010 and 2016 who were assigned to a higher risk group according to the classifications of Fletcher and Miettinen. Concerning the PDGFRA gene, a wild type was found in all cases. The KIT/exon 11 (n=6) was most frequently affected by mutations, followed by KIT/exon 9 (n=2) and 18 (n=1).

**Table 3:** Results of the mutation analysis of gastrointestinal stromal tumors (GIST) in relation to localization, risk classification, proliferation rate and clinical course.

Mutation	Location GIST	Metastasis	Secondary malignancy	Classification according to Fletcher	Classification according to Miettienen	Proliferation rate	Therapy with Imatinib	Recurrence/progress in the course	Dying from GIST	Follow-up (months)
**Exon 11**	Jejunum	No	No	High risk	High risk	15%	Yes	Recurrence	No	81
**Exon 11**	Stomach	No	No	High risk	Intermediate risk	15%	Yes	Recurrence	No	84
**Exon 11**	Stomach	Yes	Yes	High risk	High risk	15%	Yes	Progress	Yes	13
**Exon 11**	Jejunum	Yes	Yes	High risk	High risk	8%	Yes	Progress	Yes	57
**Exon 11**	Ileum	No	No	High risk	High risk	10%	Patient refused	Recurrence	Yes	21
**Exon 9**	Ileum	No	No	Intermediate risk	High risk	1%	Yes	No	Death from another cause	6
**Exon 9**	Jejunum	No	Yes	High risk	High risk	2%	Yes	No	No	58
**Exon 11**	Stomach	Yes	No	High risk	High risk	20%	Yes	No	No	38
**Exon 18**	Stomach	Yes	No	Low risk	Intermediate risk	20%	Yes	Progress	Yes	36

### Prognostic value of the proliferation rate

In 56 of the 58 patients (97%), the pathological findings reported the proliferation rate using the proliferation marker MIB 1. The proliferation rate (Tables 2 and 4) varied between 0 and 70% (mean value 5%). We divided the group of 21 patients who developed a second malignancy during the study period into patients with tumors with a low (<5%) or high proliferation rate (≥5%), depending on the proliferation rate (Table 4). Using a four-field test, we tested whether the proliferation rate had a significant influence on the occurrence of a second malignancy. However, the evaluation in the four-field test at the significance level of 0.05 showed no influence of the proliferation rate on the occurrence of second malignancies (p=1.0).

**Table 4:** Proliferation rate of non-metastatic gastrointestinal stromal tumors (GIST) in relation to size and location.

Localisation of GIST	Tumor size	Proliferation rate		
		<5%	5–10%	>10%
**Stomach (n=26)**	<2 cm	8	1	0
	2–5 cm	7	2	0
	>5 cm	4	2	2
**Small bowel (n=23)**	<2 cm	7	0	0
	2–5 cm	10	1	0
	>5 cm	2	2	1
**Rectosigmoid (n=1)**	<2 cm	0	0	0
	2–5 cm	1	0	0
	>5 cm	0	0	0

A total of seven patients died of GIST. Four of these patients had a metastatic tumor, and three had a small bowel GIST with a size of more than 5 cm and a proliferation rate >5%. The remaining patients survived recurrence-free until the end of the study.

While only a few primarily non-metastatic GIST showed a proliferation rate of ≥10% (Table 5), the majority of patients with hepatic metastases showed a significantly increased proliferation rate (Table 2).

**Table 5:** Characteristics and courses of primarily non-metastatic gastrointestinal stromal tumors (GIST) with a proliferation rate ≥10%.

Proliferation rate	10%	10%	15%	15%
**Localisation of GIST**	Ileum	Stomach	Stomach	Jejunum
**Size of GIST (cm)**	5.2	3.5	12	7
**Mitoses**	8/50 HPF*	5/10 HPF*	5/50 HPF*	11/50 HPF*
**Classification according to Fletcher**	High risk	High risk	High risk	High risk
**Classification according to Miettinen**	High risk	Intermediate risk	Intermediate risk	High risk
**Mutation**	Exon 11	No analysis done	Exon 11	Exon 11
**Therapy with Imatinib**	Rejected	No	Yes	Yes
**Recurrence**	After 20 months	No	After 59 months	After 62 months
**Follow-up**	Deceased on GIST after 21 months	72 months without recurrence	84 months without recurrence	81 months without recurrence

* – high power field.

### Follow-up care, further therapy, and mortality

Postoperative therapy with imatinib was carried out in ten (17%) patients. There was a patient with peritoneal carcinosis with malignant ileum GIST who died of pancreatic cancer two years after the initial diagnosis of GIST. Another patient received therapy with imatinib for hepatic metastatic gastric GIST (mutation analysis showed an exon 18 mutation) (Table 3). This patient died three years after the initial diagnosis with tumor progression. Imantinib therapy was recommended for eight other patients based on proven exon 9 (n=2) or 11 mutations (n=6) (Table 3). The therapeutic approach in four of these patients was palliative. Three patients died in the further course (after 13, 21, and 57 months, respectively), and one patient lived in complete remission (follow-up 58 months) (Table 3). Systematic follow-up was recommended for the remaining 48 patients (83%).

### Results of univariate and multivariate analysis of risk parameters

**Figure 5:**
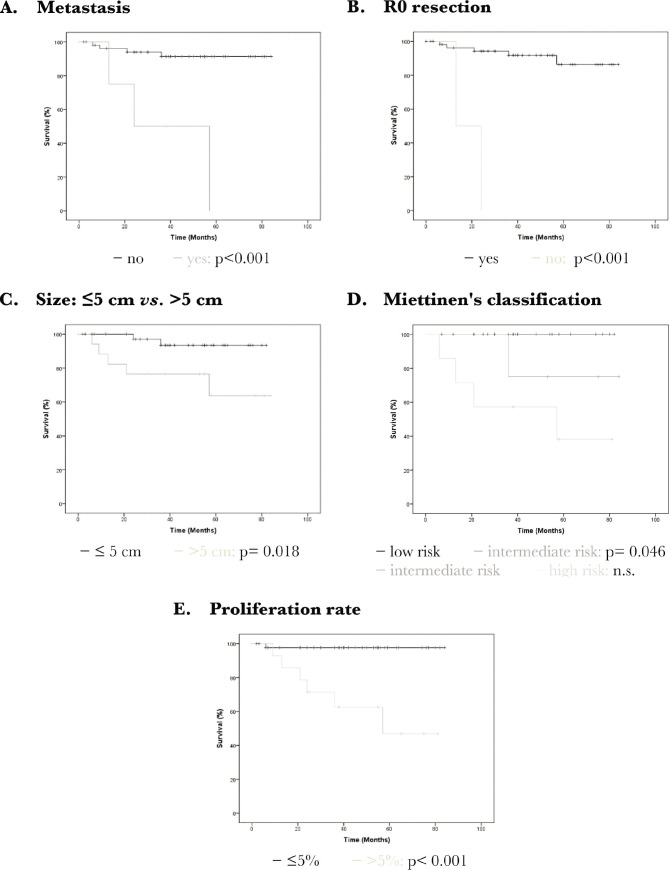
Death from gastrointestinal stromal tumors (GIST): Kaplan Meier curves as a representation of the significant parameters arising in the univariate analysis. A – Presence of distant metastasis has a significant impact on the postoperative outcome (p<0.001); B – Technical impossibility of a R0 resection has a significant impact on the postoperative outcome (p<0.001); C – A tumor size >5 cm has a significant impact on the postoperative outcome (p=0.018); D – Miettinen's classification showed a significant poorer prognosis for intermediate risk compared to low risk (p=0.046); E – A proliferation rate >5% has a significant impact on the postoperative outcome (p<0.001).

**Figure 6:**
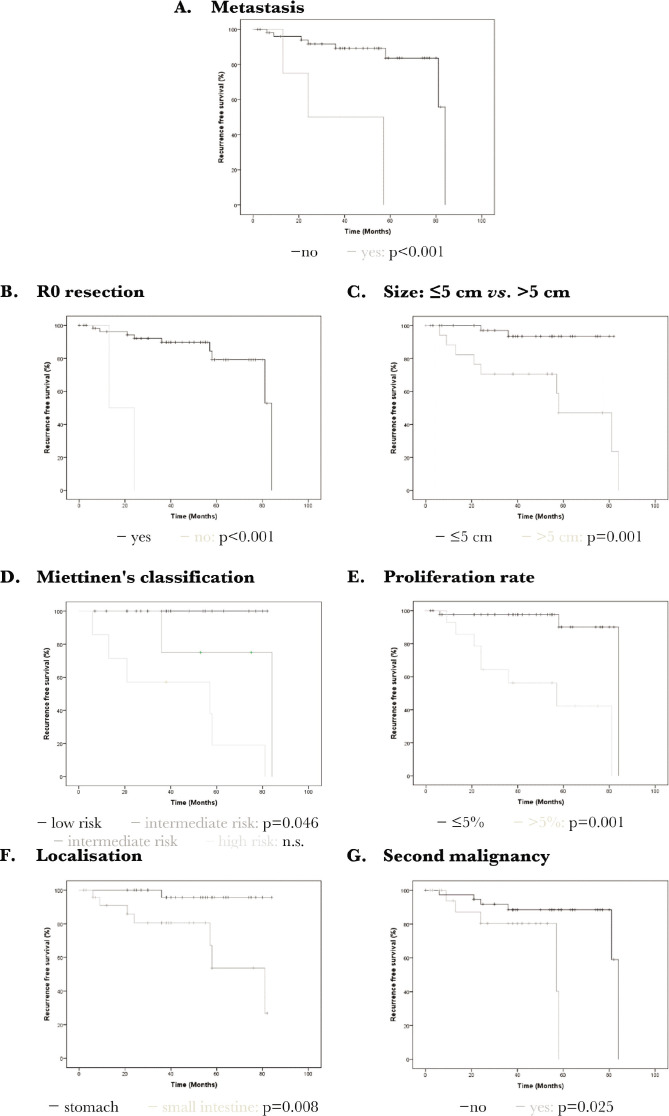
Recurrence of gastrointestinal stromal tumors (GIST): Kaplan Meier curves as a representation of the significant parameters arising in the univariate analysis. A – Presence of distant metastasis has a significant impact on recurrence (p<0.001); B – Technical impossibility of a R0 resection has a significant impact on recurrence (p<0.001); C – A tumor size >5 cm has a significant impact on recurrence (p=0.001); D – Miettinen's classification showed a significant higher recurrence rate for intermediate risk compared to low risk (p=0.046); E – A proliferation rate >5% has a significant impact on recurrence (p<0.001); F – The location of GIST in the small intestine (compared to the stomach) has a significant impact on recurrence (p=0.008); G – The presence of a second malignancy has a significant impact on recurrence (p=0.025).

The univariate analysis revealed the following risk factors for a poor prognosis in terms of death from GIST: metastasis at the time of the operation, the impossibility of R0 resection, the intermediate risk *versus* low risk following Miettinen's classification, proliferation rate >5% and tumor size >5 cm (Table 6, Figure 5 A–E). With regard to the risk of recurrence, the following parameters were also significant: localisation in the small intestine (*versus* stomach) and presence of a second malignancy (Table 6, Figure 6 A–G). In the multivariate analysis, the following parameters could be determined as significant risk factors for both deaths from GIST and risk of recurrence: Metastasis at the time of the operation, the impossibility of R0 resection, proliferation rate >5%, and tumor size >5 cm. With regard to the risk of recurrence, the localization is also significant (Table 7).

**Table 6:** Univariate analysis of risk parameters for unfavorable outcome in patients with gastrointestinal stromal tumors (GIST).

	Death from GIST (p)	Recurrence of GIST (p)
**Gender**	0.07	0.26
Female		
Male		
**Age**	0.3	0.19
<50 years		
>50 years		
**Localisation**	0.065	**0.008**
Stomach vs. small intestine		
**Metastasis**	**<0.001**	**<0.001**
Yes		
No		
**R0 resection**	**<0.001**	**<0.001**
Yes		
No		
**Size**		
≤5 cm vs. >5 cm	**0.018**	**0.001**
**Flechter's classification**		
Low risk vs. intermediate risk	0.241	0.241
Intermediate risk vs. high risk	0.854	0.71
**Miettinen's classification**		
Low risk vs. intermediate risk	**0.046**	**0.046**
Intermediate risk vs. high risk	0.313	0.107
**Proliferation rate**	**<0.001**	**<0.001**
≤5%		
>5%		
**Second malignancy**	0.06	**0.025**
Yes		
No		

**Table 7:** Multivariate analysis of risk parameters for unfavorable outcome in patients with gastrointestinal stromal tumors (GIST).

Death from GIST (p)	HR	95% CI	p
**Metastasis**	0.308	0.145; 0.652	0.007
**R0 resection**	0.217	0.088; 0.536	0.001
**Proliferation rate**	0.232	0.080; 0.668	0.007
**Size ≤5 cm vs. >5 cm**	0.174	0.034; 0.898	0.037
**Recurrence of GIST (p)**	**HR**	**95% CI**	**p**
**Metastasis**	0.344	0.168; 0.704	0.004
**R0 resection**	0.244	0.104; 0.576	0.001
**Proliferation rate**	0.284	0.130; 0.617	0.001
**Size ≤5 cm vs. >5 cm**	0.73	0.09; 0.603	0.015
**Location: Stomach vs. small intestine**	0.097	0.012; 0.799	0.03

HR – Hazard ratio; CI – confidence interval.

## Discussion

Various scoring systems [2–7] have been developed over the last 25 years to assess the clinical course of GIST. In addition to tumor size and location, these scoring systems are also based on histopathological features such as the mitotic rate. Patients with GIST, who are classified as “intermediate” or “high” risk tumors according to the current classifications, benefit from therapy with tyrosine kinase inhibitors [1, 23, 24]. Analysis of the presence of mutations in the KIT or PDGFRA gene enables conclusions to be drawn about the prognosis and response to therapy with tyrosine kinase inhibitors in these patients [18, 25–27].

Discrepancies between the assessment of the biological behaviour of GIST according to the current classifications by Fletcher [2] and Miettinen [6] and the actual clinical course were found in our patient collective. The aim of the present study was, therefore, to check the benefits of the above-mentioned scoring systems in everyday clinical practice. It is crucial for the clinician to be able to apply the risk stratifications created on the basis of theoretical classifications in practice. The following questions are posed to the clinician: Is the risk of metastasis and recurrence of GIST classified as low-risk tumors so low that regular follow-up care is not necessary? Are there parameters that can predict the risk of possible secondary tumors? Are there criteria that speak in favor of carrying out appropriate examinations to find possible secondary tumors when diagnosing a GIST? Can aftercare planning or further therapy planning after resection of GIST be based on the classification, or is the risk of metastasis or recurrence higher than expected?

The study aimed, therefore, to examine in our patients collective to what extent the classifications correlate with the clinical course. Based on the determination of the proliferation rate, it should be evaluated whether it is useful to add further parameters to the classifications according to Fletcher [2] or Miettinen [6] in order to enable a more realistic assessment of the probability of metastasis, risk of recurrence and correlation with secondary tumors.

### Operation and perioperative course

About 30% of GIST diagnoses are incidental findings made during other operations [28]. The present study supports this observation with an even larger proportion of 49% (n=19) of GIST discovered as an incidental finding. In the therapy of localized GIST, the focus is on surgical R0 resection [16]. We achieved R0 resection in 56 of 58 patients (97%). As there is no need for systematic lymph node dissection and a safe distance of 2 cm is sufficient, a laparoscopic approach is possible for smaller localized findings [22, 29]. For gastric GIST, however, this was only done in four of 21 patients (19%) in our patient collective. In the remaining patients, a laparoscopic procedure was not possible due to the underlying disease leading to the operation, the extent of the GIST, and existing adhesions. Postoperative GIST-specific complications did not occur in the examined patient group after resection of incidentally diagnosed small and locally limited GIST. The perioperative morbidity and mortality of the patients were not influenced by the additional diagnosis of GIST. Complications occurred in 2 patients on whom the surgical procedure was primarily due to a GIST. These were an anastomotic leak after proximal gastrectomy and death in hemorrhagic shock after right hemihepatectomy.

### Coincidence with secondary malignancies

In the literature, a coincidence rate with second malignancies of 14–43% is reported [12, 30]. Our data also showed a coincidence of GIST with other neoplasms. In our investigations, six of the 58 patients (10%) had synchronous benign tumors. 20 of the patients (34%) suffered synchronously or metachronically from malignant neoplasia.

### Classification according to mitotic rate

In 1998 the pathophysiological importance of KIT for the development of GIST was first described. However, risk classifications of soft tissue tumors already existed before. The most cited classification was taken from the work of Franquemont [3, 31], who already used the tumor size, mitotic rate, and proliferation rate to differentiate between low-risk and high-risk tumors. In retrospect, however, there was no differentiation between stromal and smooth muscle tumors.

Since 2002, the risk stratification of patients with GIST has initially been based on the Fletcher classification. According to Fletcher's recommendation, the following parameters were named as the most important tumor characteristics and disease-defining factors at a consensus conference: positivity to CD 117, tumor size, and mitotic rate [2].

In 2006, the research group headed by Miettinen and Lasota showed that, in addition to the mitotic rate and tumor size, the localization of the tumor provides significant information about the malignancy potential of the tumor [6]. The tumor nodes metastases (TNM) classification created by Wittekind *et al*. [7] for GIST in 2010 is currently the latest classification. The TNM classification supplements the classification of Miettinen and Lasota by including lymph node or distant metastases. These automatically lead to a classification of the GIST in Union Internationale Contre le Cancer (UICC) Stage IV.

In all classifications established after 1998, the mitotic rate is mentioned as a prognostic criterion. However, there are no standardized procedures where the mitoses should be counted, how they should be counted, and how large 50 HPF should be. According to the experience of Agaimy *et al*. [30], too many mitoses are often counted. The reasons given are irregular lymphocytes and other inflammatory cells between the tumor cells, as well as so-called apoptotic bodies in GIST cells. In addition, many GIST show a heterogeneous distribution of the mitotic rates in the tumors so that it can be decisive where in the tumor tissue the mitoses are counted.

As part of our work, the mitotic rate of GIST was determined in 37 patients. In this way, it was possible to assign these tumors to the Fletcher or Miettinen risk classification. A review of the patients who died due to the progression of the GIST or who had already metastasized at the time of the operation or diagnosis of the GIST, made it clear that in one case, the risk potential of a GIST would have been clearly undergraded according to the current classifications. This example makes it clear that with GIST that are histopathologically classified as low, there is a risk of overlooking possible distant metastases. Without adequate staging and adequate follow-up care, the aggressiveness of the tumor can easily be underestimated in these cases.

### Proliferation rate as a prognostic factor

In our study, we examined the monoclonal antibody MIB 1 as a marker for the proliferation activity of GIST cells in 56 of 58 patients (97%) [32]. The proliferation rate is given as the number of MIB1 positive cells per 100 tumor cells. There is disagreement in the literature about whether to recognize the proliferation marker MIB 1 as a good indicator of the risk of recurrence or metastasis. In the study published by Carrillo *et al*. [33] in 1997, the proliferation rate was presented as a good independent parameter for assessing the biological behavior of GIST. Wong *et al*. [34], on the other hand, questioned the informative value of the proliferation rate and identified in their retrospective study the mitotic rate as the most important prognostic factor. In 2005 Ohdaira *et al*. published their data from 135 patients with GIST, in which they identified the tumor size (>5 cm) and the proliferation rate (≥40/mm^2^) as significant prognostic factors [35]. More recent studies [36, 37] also support the value of the proliferation rate as an independent predictive value for relapse-free survival of GIST patients. Also, the data published by Belev *et al*. [36] show that there is no significant difference in the proliferation rate between gastric GIST and small bowel GIST. From this, they concluded that the proliferation rate can be assessed as a non-site-specific prognostic factor. Wen-Yi Zhao's working group [37] divided the patients classified as high-risk according to modified National Institutes of Health (NIH) criteria [5] into 3 groups according to their proliferation rate (≤5%, 6–8%, and >8%) and showed that a proliferation rate>8% significantly increases the risk of recurrent disease. In our patient collective, the proliferation rate was also of prognostic relevance in the multivariate analysis. With regard to death from GIST, a proliferation rate >5% in addition to a tumor size >5 cm, metastasis at the time of surgery, and the impossibility of R0 resection were associated with significantly poorer survival. Assessing the outcome of our patients with regard to the recurrence rate, the location of the tumor in the small intestine (*versus* the stomach) was another significant parameter.

According to our results, 39 (78%) of the total 50 GIST who were not metastatic at the time of surgery had a proliferation rate of <5%. A proliferation rate between 5 and 10% was found in 8 tumors (16%). In three cases (6%), the primary proliferation rate was >10% (Table 4). A significant increase in the proliferation rate in liver metastases from GIST was noticeable. In our collective, the proliferation rates in the histopathologically examined liver metastases were between 10 and 70% (Table 2). In the above-mentioned patient with a 3 cm gastric GIST and synchronous liver metastasis, the proliferation rate of the liver metastasis was 20%, while the proliferation rate in the primary was only 1%. In this patient, determining the primary proliferation rate would not have provided any information on the malignant biological behavior of this GIST (Table 2).

Even if the mitotic rate was not determined, the metastatic bowel GIST, according to Miettinen, were rated as high-risk tumors based on their size. This reflects the actual clinical course well. The proliferation rate of these tumors was 8% and was thus significantly increased compared to the majority of GIST examined (Table 2).

In our study, we also examined a possible correlation between the proliferation rate and the incidence of secondary malignancies. In 20 of the 21 patients in our collective with secondary malignancies, the GIST proliferation rate was determined using MIB 1. A MIB 1 ≥5% was found in only six cases. In seven tumors, the proliferation rate was below 5%, and in the remaining seven, even below 1%. Of these 14 tumors, 12 cases were assumed to be less aggressive (low risk). Only two patients with the localization of the GIST in the jejunum were assigned to the intermediate or high-risk group due to the tumor size of 4.8 and 10.7 cm, respectively.

Thus the proliferation rate in 70% of the patients with associated secondary malignancies was less than 5%. This underlines the assumption that the malignancy potential of GIST does not indicate the synchronous or metachronous occurrence of a malignant tumor of a different dignity. The evaluation in the four-field test confirmed no significant influence of the proliferation rate on the coincidence of secondary malignancies.

## Conclusion

With an incidence of 1.3–2 cases per 100000 population, GIST are relatively rare tumors. Of the diagnosed GIST, surgical resection is only indicated for tumors >2 cm. GIST are very often incidental findings diagnosed during other operations. These are mostly operations due to malignancies of other dignities. Perioperative morbidity and mortality are not influenced by the additional diagnosis of a locally limited GIST. In the case of small GIST, a minimally invasive procedure, such as a laparoscopic resection, should be sought. Patients with GIST showed a striking frequency of coincidences with other benign or malignant tumors. However, no assessment of the probability of the occurrence of a secondary neoplasm can be derived from the common risk classifications of GIST. The determination of the proliferation rate in the GIST cells does not prove to be additionally helpful in this regard. Another problem when dealing with GIST classified as low risk using the current classifications is the fact that metastatic growth cannot be ruled out with certainty. In this regard, too, the proliferation index does not allow the prediction of actual biological behavior and prognosis to be specified more precisely.

The power of our study is limited by the low sample size of 58 patients. However, with regard to the rarity of GIST requiring surgery, our study shows a relatively large patient collective. Meta-analyses and multicentre studies represent an option to better assess the prognostic value of classifications and prognosis parameters with regard to the postoperative outcome of this rare tumor entity.

## Data Availability

For national data protection reasons, it is not possible to pass on the data without obtaining corresponding consent.
